# Effects of Cooking Method on the Antioxidant Activity and Inhibition of Lipid Peroxidation of the Javanese Salad “Pecel” Vegetables and Its Peanut Sauce Dressing

**DOI:** 10.1155/2021/8814606

**Published:** 2021-02-18

**Authors:** Gregorius Tsiompah, Retno Murwani, Nani Maharani

**Affiliations:** ^1^Department of Nutrition-Master Program in Nutrition Science, Faculty of Medicine, Universitas Diponegoro, Semarang, Indonesia; ^2^Faculty of Animal and Agricultural Sciences, Universitas Diponegoro, Semarang, Indonesia; ^3^Natural Product Laboratory-UPT-Laboratorium Terpadu (Integrated Laboratory for Research and Services), Universitas Diponegoro, Semarang 50275, Indonesia; ^4^Department of Pharmacology and Therapy, Faculty of Medicine, Universitas Diponegoro, Semarang 50275, Indonesia

## Abstract

Vegetables are essential in our diet to maintain health, partly due to their antioxidant properties. A well-known Javanese salad called “Pecel” is prepared by boiling the vegetables and dressed with seasoned peanut sauce. Cooking can reduce or improve the antioxidant properties of foods; therefore, the purpose of this study was to evaluate the effects of brief water boiling (1 min), steaming (1 min), and water blanching (20 s) of the Javanese Pecel vegetables, with or without the peanut sauce. We assessed the *in vitro* antioxidant capacity and lipid peroxidation inhibition of the salad samples prepared using each cooking method. Six vegetables, i.e., *Sesbania grandiflora* (turi) flower, *Amaranthus hybridus* L. (spinach), *Carica papaya* (papaya) leaves, *Cosmos caudatus L*. (kenikir) leaves, *Vigna unguiculata ssp. Sesquipedalis* (yard-long beans), and *Vigna radiata* (mung-bean) sprouts were cooked by boiling or steaming for 1 min or blanching for 20 s. Peanut (*Arachis hypogaea*), the raw material for peanut sauce, was fried in either fresh palm oil or repeatedly used palm oil. Our results revealed that the highest antioxidant capacity (percent inhibition of DPPH radicals) was observed following boiling for 1 min in case of spinach (41.94 ± 9.8%), papaya (59.04 ± 5.35%), kenikir (54.93% ± 6.32%), and yard-long beans (70.21 ± 8.91%); steaming for 1 min in case of turi flower (60.25 ± 3.63%); and blanching for 20 s in case of mung-bean sprouts (49.27 ± 3.69%). Peanut sauce prepared by frying peanuts in fresh or repeatedly used palm oil reduces the natural antioxidant and lipid peroxidation inhibition properties. However, seasoning the peanut sauce with fresh garlic and lime leaves can restore the lost antioxidant properties. Our study provides the first and clear evidence of the optimal cooking method for Pecel vegetables and sheds light on the wisdom behind the existing traditional cooking method.

## 1. Introduction

Vegetables are well-known key components of the human diet and are beneficial because of the presence of diverse phytonutrients, such as polyphenols, flavonoids, and carotenoids, which have strong antioxidant properties [1–3]. These ameliorate, prevent, or reverse several undesirable effects of metabolic syndromes, such as dyslipidaemia, oxidative stress status, hyperglycaemia, and obesity [4–7]. Green leafy vegetables, the flowery part of vegetables, and/or the sprouts of some beans have been part of the daily diet in Southeast Asian countries. A traditional Javanese (people living in Java island of Indonesia) salad called “Pecel” is one of the oldest known salad dishes in Indonesia and has been consumed for decades. It consists of a variety of cooked vegetables dressed with seasoned peanut sauce [8]. The vegetables are procured locally, and the ingredients of the salad vary according to the seasonal availability. Although Pecel has been a part of the daily diet of Javanese people, no data on its consumption and production are available. Pecel salad is also sold commercially in diverse outlets, ranging from individual street vendors to restaurants and online vendors, in all parts of the Java island. It is also available in many parts of other Indonesian islands, even in overseas Indonesian restaurants.

Pecel is prepared by boiling the vegetables briefly and dressing them with a seasoned peanut sauce. The vegetables most commonly used in Pecel consist of *Sesbania grandiflora* (turi) flower, *Carica papaya* (papaya) leaves, *Cosmos caudatus L.* (kenikir) leaves, *Amaranthus hybridus L.* (spinach), *Vigna unguiculata ssp. Sesquipedalis* (yard-long beans), and *Vigna radiata* (mung-bean) sprouts. Boiling each type of vegetable alternately using the same aluminium kitchen pot is the general practice, because of its simplicity and swiftness in preparation. From the food safety point of view, this practice inactivates microbial residues after vegetable washing. The dish can be consumed with or without steamed rice and protein-rich foods, such as cooked “tempe” (fermented soybean) or “tahu” (tofu), chicken egg, vegetable crackers, or a combination of a few of them. As one of the Javanese people, we (the author) have been consuming Pecel as a part of our daily diet since childhood.

The practice of cooking Pecel vegetables by boiling may enhance their antioxidant properties depending on the cooking method and duration [4, 9–13]. The seasoned peanut sauce dressing, which is mixed just before consumption, is prepared by deep frying the peanut in palm oil. Oil is drained, and the peanut is mixed with garlic, lime leaves, and a pinch of palm sugar and salt and ground together to be used as the dressing. There have been concerns regarding peanut sauce, because of the scientific knowledge on the potential side effects. Many studies have shown that using cooking oil repeatedly results in production of hazardous radicals such as peroxides and polar compounds (Murwani, unpublished data) [14]. Long-term consumption of foods prepared using reheated oil could severely hamper the antioxidant defence network, leading to pathologies such as hypertension, diabetes, and vascular inflammation [13, 15, 16].

It is important to understand the effect of cooking methods on the antioxidant activity of vegetables and the dressing sauce. Therefore, this study was carried out to evaluate the effect of different cooking methods (boiling, water steaming, and water blanching) on the antioxidant capacity of Pecel vegetables. Furthermore, the antioxidant and lipid inhibition capacities of cooked and mixed Pecel vegetables before and after dressing with seasoned peanut sauce were evaluated *in vitro*.

## 2. Materials and Methods

### 2.1. Vegetables and Chemicals

**Figure 1:**
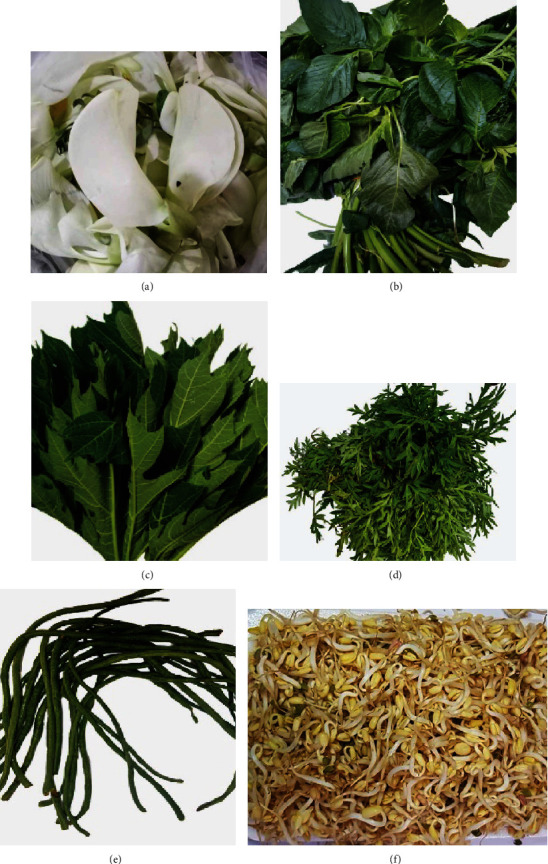
Fresh raw vegetables for Pecel. (a) *Sesbania grandiflora* (turi) flower, (b) *Amaranthus hybridus* L. (spinach), (c) *Carica papaya* (papaya) leaves, (d) *Cosmos caudatus L*. (kenikir) leaves, (e) *Vigna unguiculata ssp. Sesquipedalis* (yard-long beans), and f. *Vigna radiata* (mung-bean) sprout.

Fresh vegetables, i.e., *S. grandiflora* (*turi*), *C. papaya* (papaya) leaves, *C. caudatus L.* leaves, *A. hybridus L.* (spinach) leaves, *V. unguiculata ssp. Sesquipedalis* (yard-long beans), and *Vigna radiata* (mung-bean) sprouts, *Citrus hystrix DC.* (lime) leaves, *Arachis hypogaea* (peanut), *Allium sativum* (garlic), palm sugar, and salt were obtained from the local market.  Figure 1 shows the vegetables used to prepare Pecel since ancient times. All chemicals and solvents used were of analytical grade. *2.2-diphenyl-1-picrylhydrazyl* (DPPH), absolute (96%) ethanol, chicken egg yolk, *phosphate buffer* (pH 7.4), ferrous sulphate (FeSO_4_), trichloroacetic acid (TCA), and thiobarbituric acid (TBA) were obtained from Sigma-Aldrich.

### 2.2. Pecel Vegetable Preparation

The vegetables were cleaned under running water and drained. Edible parts/portions were collected, were weighed (EPW), sliced, and mashed using a blender, for analyses. Fresh peanuts were also mashed to analyse their antioxidant activity.

### 2.3. Cooking Methods

All cooking was done using household kitchen utensils, i.e., aluminium pot and steamer with 25 cm diameter and 15 cm depth. Each type of vegetable was cooked by boiling for 1 min, steaming for 1 min, or water blanching for 20 s. The time duration was based on a preliminary observation and survey from some of the Pecel outlets (street vendors and restaurants). Our observation was that Pecel vegetables were cooked for a maximum of 2 min and the mung-bean sprouts for a minimum of 20 s. It is also based on a study that found cooking of vegetables for less than five minutes maintains a flavonol content of more than 80% [17]. Based on these information, we ran a quick test (one single data) for cooking time of up to 5 min and found that one-minute boiling and steaming, for several Pecel vegetables, and 20 s blanching for bean sprouts were the best for retaining the antioxidant properties. People cooking in households and street vendors do not measure the exact cooking time of Pecel vegetables; they instead cook based on a long practical experience.

### 2.4. Boiling

Approximately 100 g of each type of Pecel vegetable (edible portion weight after cleaning, washing, and draining) was added to boiling water (1200 mL; 100°C) in a covered aluminium kitchen pot (diameter, 25 cm; height, 15 cm) and occasionally stirred to ensure even cooking of the vegetables. The vegetables were completely submerged in the boiling water. After 1 min, the vegetables were removed, drained, cooled to room temperature, and mashed using a blender for analyses.

### 2.5. Steaming

Approximately 100 g of each type of vegetable (edible portion weight after cleaning, washing, and draining) was placed on a perforated tray in an aluminium kitchen-steamer (diameter, 25 cm; height, 15 cm) that contained boiling water (1200 mL; 100°C). The boiling water was below the perforated tray. During the steaming, the lid was closed, and the vegetables were occasionally stirred to ensure even cooking of the vegetables. After 1 min, the vegetables were removed, cooled to room temperature, and mashed using a blender for analyses.

### 2.6. Blanching

About 100 g of each type of vegetable (edible portion weight after cleaning, washing, and draining) was added to boiling water (1200 mL; 100°C) in an uncovered aluminium kitchen pot. All vegetables were submerged in the boiling water. During the blanching process, the vegetables were stirred to ensure cooking. After 20 s, the vegetables were removed, cooled to room temperature, and mashed using a blender for analyses.

### 2.7. Peanut Frying

A total of 250 g of fresh peanuts were added to 500 mL of palm oil in an aluminium frying wok at 100–120°C and fried until golden brown. The fried peanuts were removed, drained, and cooled to room temperature. The peanuts were deep-fried using either fresh palm oil, hereinafter referred to as “first oil (FO)-fried peanuts” or with palm oil that was already used five times for frying, hereinafter referred to as “repeatedly used oil (RO)-fried peanuts.”

### 2.8. Peanut Sauce

**Figure 2:**
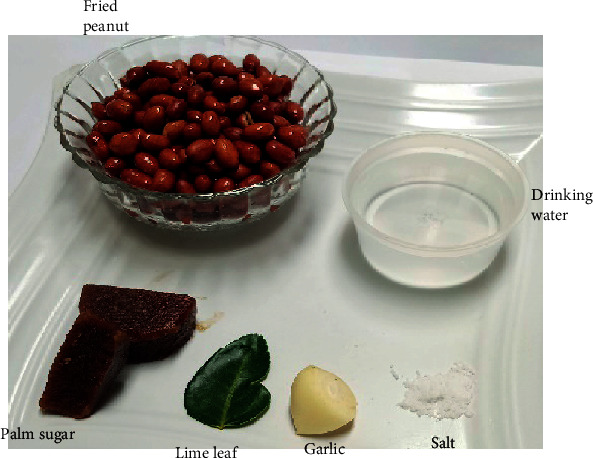
Peanut sauce ingredients: fried peanut, drinking water, garlic, lime leaf, and palm sugar.

The ingredients for peanut sauce were fried peanut, water, garlic, lime leaf, and palm sugar (Figure 2). Although chili can be added as an ingredient to satisfy customers, who are fond of hot spicy sauce (varying degrees), here, we used the basic ingredients without chili. A total of 250 g of fried peanuts was mashed using a blender. About 50 g of peanut was added to 5 g of fresh garlic, 1 fresh lime leaf, and 30 g of palm sugar (Figure 2). The mixture was mashed using a masher, followed by the addition of warm water and salt to taste. Two kinds of peanut sauce were made using either FO- or RO-fried peanuts and designated as FO and RO peanut sauce, respectively.

### 2.9. Combined Cooked Vegetables (Pecel Vegetables)

From a total of 100 g of each of the six cooked vegetables, 25 g of each of the vegetable was weighed, mixed in a ratio of 1 : 1 (a total of 150 g), and mashed using a blender for analyses. In the normal consumption practice, cooked vegetables are consumed without being mashed.

### 2.10. Pecel Salad (Combined Cooked Vegetables with Peanut Sauce)

**Figure 3:**
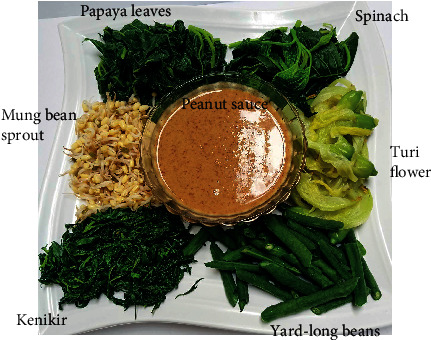
The cooked Pecel vegetables and peanut sauce are ready to be consumed by mixing/dressing the vegetables with the peanut sauce. In this study, the cooked vegetables and peanut sauce were homogenised using a kitchen blender for analyses.

The six cooked vegetables (Figure 3) were mashed as described above and blended with 150 g of peanut sauce for analysis.

We considered two treatments: (1) combined vegetables mixed with FO peanut sauce, hereinafter referred to as “Pecel FO,” or (2) combined vegetables mixed with RO peanut sauce, hereinafter referred to as “Pecel RO.” The mixture was homogenised using a kitchen blender for analyses. In practice, cooked Pecel vegetables and its peanut sauce are mixed just before consumption, similar to a western salad.

### 2.11. Determination of Antioxidant Activity by the DPPH Method

DPPH is a stable free radical that has a deep violet colour in methanol or ethanol and has maximum absorbance at 517-520 nm. When an antioxidant neutralises DPPH, the solution becomes colourless or pale yellow and absorbance is reduced. As antioxidant activity increases, the absorbance of the DPPH solution decreases. The antioxidant activity of samples (Pecel vegetables, peanuts, etc.) was evaluated according to the method described by Rezaie et al. [18] and optimised by Murwani (unpublished) using 60 *μ*M DPPH in ethanol (control radicals). We obtained the maximum absorbance for the DPPH solution at 517 nm. Antioxidant activity was determined by adding 0.1 g of each sample to 5 mL of 60 *μ*M DPPH. The reaction mixture was homogenised using a vortex and incubated for 1 h at room temperature in the dark. The absorbance of the mixture was recorded against a blank (ethanol) at 517 nm using a UV-Vis spectrophotometer (Spectroquant®Pharo300). This absorbance is designated as sample absorbance (*A*_sample_), and each sample was analysed in triplicate. Antioxidant activity was expressed as percent inhibition of DPPH radicals using the following equation:

(1) $$\begin{array}{c} \text{Inhibition }\left(\%\right)=\left[\left(A_{\text{control}}–A_{\text{sample}}\right)/A_{\text{control}}\right]\times 100, \end{array}$$

where *A*_control_ is the absorbance of the 60 *μ*M DPPH solution and *A*_sample_ is the absorbance of the sample.

### 2.12. Determination of Lipid Peroxidation Inhibition

Lipid peroxidation inhibition of egg yolk was performed according to the method described by Rahman et al. in 2015 [19]. Briefly, 0.1 g of sample was added to 0.5 g of chicken egg yolk emulsified with 0.1 M phosphate buffer (pH 7.4) to obtain a final concentration of 25 g/L. The mixture was added to 100 mL of 1 M FeSO_4_ to induce lipid peroxidation. The mixture was shaken vigorously and incubated at room temperature for 1 h. Next, 0.5 mL of 15% TCA and 1 mL of thiobarbituric acid (TBA) were added to the mixture and incubated in a boiling water bath for 10 min. The tube containing the reaction mixture was cooled to room temperature and centrifuged at 3500 g for 10 min to precipitate the proteins. The supernatant was sampled to measure the formation of TBA reactive substances by recording the absorbance at 532 nm using a UV/Vis spectrophotometer (Spectroquant®Pharo300). A mixture of phosphate buffer, egg yolk, and FeSO_4_ without the sample was used as the control. The analysis was carried out in triplicate. The percentage of lipid peroxidation inhibition was calculated using the following formula:

(2) $$\begin{array}{c} \text{Inhibition }\left(\%\right)=\left(A_{0}–A_{S}\right)/A_{0}\times 100, \end{array}$$

where *A*_0_ is the absorbance of the control and *A*_*S*_ is the absorbance of the sample from the reaction mixture containing egg yolk, phosphate buffer (pH 7.4), FeSO_4_, 15% TCA, and TBA.

### 2.13. Data Analysis

All data are presented as the mean ± SD (*n* = 3). The data were tested for normality using the Shapiro–Wilk test (*n* < 50). Normally distributed data were further tested using a one-way ANOVA and an independent *t*-test. The difference between the means was further analysed using the smallest significance difference at 95% confidence (*p* < 0.05). The statistical analyses were performed using SPSS 15.

## 3. Results and Discussion

### 3.1. Effect of Cooking Method on the Water Content of Pecel Vegetables

The water content of the cooked Pecel vegetables is shown in Table 1. The results show that the cooking method (boiling, steaming, and blanching) can significantly affect the water content of Pecel vegetables (*p* > 0.05) for turi flower, spinach, papaya, and kenikir leaves. However, it does not affect the moisture content of yard-long beans and mung-bean sprouts.

The water content of turi flower decreased significantly by approximately one percent, after cooking using any of the three methods, while that of spinach and papaya decreased significantly with blanching and steaming. The moisture content of kenikir leaves decreased when they were blanched, but increased significantly when they were steamed. The difference in water content of cooked vegetables depends on the water absorption by vegetables during cooking. Cooking can increase or decrease the moisture content of cooked vegetables depending on the type of vegetables and cooking method. An increase in the moisture content of vegetables is generally related to higher fibre content and other chemical components of vegetables [9]. Our results demonstrated that the water content of Pecel vegetables, cooked by boiling, steaming, or blanching varied depending on the type of the vegetable. We selected cooked Pecel vegetables (underlined) based on their antioxidant activity (Table 2) and significant retention of moisture content compared to that of the raw vegetables (*p* < 0.05) (Table 1).

### 3.2. Cooking Methods of the Individual Vegetables of Pecel and Their Antioxidant Activity

The antioxidant activity of each Pecel vegetable is shown in Table 2. Differences in cooking methods significantly affected the antioxidant activity of each vegetable (*p* < 0.05). Blanching was done for a brief time, i.e., 20 s, during which some of the Pecel vegetables (spinach, papaya leaves, turi flower, and yard-long bean) were not cooked yet, except the bean sprouts. This uncooked condition of the vegetables, after blanching, reduced their antioxidant activity compared to that of the control. However, after blanching, the antioxidant activity of the bean sprout that had been cooked well increased (to 49.27%) significantly (*p* < 0.05), compared to that of the control (24.37%) (Table 2).

Boiling the Pecel vegetables for 1 min affected the degree of cooking and colour, indicating a change in texture and their phytochemical content. As shown in Table 2, boiling significantly increased the antioxidant activity of spinach (43.15%), papaya leaves (59.04%), kenikir leaves (54.93%), and turi flower (53.84%), compared to that of their respective controls (*p* < 0.05), while that of yard-long bean (70.21%) was the same as that of the control. Long yard bean has the highest antioxidant activity, as indicated by an almost instant change in the colour of DPPH solution to pale yellow, as soon as the yard-long bean sample was added to the DPPH solution. However, the antioxidant activity of the bean sprout was reduced to 10.18% after boiling when compared to that of the respective control (24.37%).

Water steaming of Pecel vegetables for 1 min affects the degree of cooking, and their colour change indicates a textural and chemical change within the vegetable matrix. The antioxidant activity of turi flowers increased significantly (*p* < 0.05) after steaming, from 33.20% when raw to 60.25% after one-minute steaming. The highest antioxidant activity was achieved after boiling for 1 min in case of spinach (43.15%), papaya leaves (59.04%), kenikir leaves (54.93%), and yard-long beans (70.21%). However, the highest antioxidant activity for turi flowers was achieved with steaming for 1 min (60.25%) and for mung-bean sprouts with blanching for 20 s (49.27%).

Moist heating increases the phenol content of vegetables. Short heat exposure softens the vegetable tissues, opens the seed coat, and facilitates the extraction of phenolic compounds and carotenoids. It could also be attributed to the possible breakdown of complex polyphenolic compounds, such as tannins to simple polyphenols, during heat processing [20, 21]. However, longer heat exposure decreases antioxidant activity, because of the partial degradation of bioactive compounds. The antioxidant activity of boiled or steamed turi flowers (*p* > 0.05) was not significantly different, although the numerical values of steamed turi flowers were higher.

Overall, it is evident from the results that brief boiling can retain the highest antioxidant activity in case of green leafy vegetables, yard-long beans, and turi flowers. Such brief cooking has been in use for decades, because of its ease and speed of preparation. Moreover, brief boiling can reduce or eliminate microbial residues from prior vegetable washing. The only vegetable that needs to be cooked separately is mung-bean sprouts; its highest antioxidant was achieved with brief blanching. Blanching is similar to boiling, but is performed for a shorter time (20 s compared to 60 s). The practice of boiling mung-bean sprouts together with other vegetables depends on the duration for which they are boiled. The sprouts can be placed on top of other vegetables in a boiling pot, removed, and drained after 20 s. Our results confirmed that cooking of Pecel vegetables increases their antioxidant activity, possibly because of the increased availability of antioxidant compounds [22, 23]. For subsequent tests of combined cooked vegetables, we selected the vegetables with the highest antioxidant values (Table 2; underlined).

### 3.3. Antioxidant Activity of Fried Peanuts, Peanut Sauce, and Pecel Salad (Combined Cooked Vegetables with Peanut Sauce Dressing)

The results show that fresh raw peanuts have significant antioxidant activity (Table 3). Antioxidant activity of the peanuts can be attributed to the flavonoids, resveratrol, vitamin E in oil, chlorogenic acid, caffeic acid, coumaric acid, ferulic acid, and possibly some other nutrients [24–26]. Deep-frying affected the antioxidant activity of peanuts, significantly (*p* < 0.05). Compared to fresh peanuts, FO peanuts had reduced antioxidant activity (9.8% reduction). This activity was further reduced in RO peanuts (16.92% reduction). The reduction in antioxidant activity could be because of the partial loss of antioxidant compounds in the heated cooking oil [16, 27].

The reduction in antioxidant activity of FO peanut or RO peanuts was compensated after they were processed into peanut sauce, with their antioxidant activity increasing significantly (34.65% to 48.07% and 27.53% to 34.77%, respectively). It is possible that the increase in the antioxidant activity of peanut sauce is likely due to the added seasonings, that is, raw garlic and lime leaves. Raw garlic and fresh lime leaves have strong antioxidant activity [28, 29].

Both the fresh seasonings used can scavenge free radicals in fried peanuts and, therefore, improve the antioxidant activity of peanut sauce. Our results suggested that to reduce or neutralise radicals from RO peanut, use of fresh garlic (not only one clove) in peanut sauce preparation is recommended.

### 3.4. Inhibition of Lipid Peroxidation by Combined Cooked Vegetables with or without Peanut Sauce

Lipid peroxidation diminishes the nutritional quality of vegetables and form toxic compounds. Antioxidants are substances that can retard the oxidation of easily oxidisable biomolecules such as lipids and proteins. Lipid peroxidation inhibition is the percent inhibition of lipid peroxidation by antioxidant compounds. The higher the lipid peroxidation inhibition, the higher is the amount of antioxidant components in the material.  Table 4 shows that the addition of FO or RO peanut sauce to the cooked vegetable mixture significantly reduced the capacity to inhibit the peroxidation of egg yolk (*p* < 0.05).

Inhibition of egg yolk peroxidation by the combined cooked vegetables was low (23.81%) (Table 4). Low inhibition can be attributed to the long boiling step (unlike brief boiling) during the determination of the lipid peroxidation inhibition method. This process could cause a series of changes in the structure of vegetable tissues, resulting in the loss of bioactive compounds, changes in the structure, and reduction in antioxidant stability [30]. The presence of iron compounds in the reaction could further reduce antioxidant activity [3].

The inhibition of egg yolk peroxidation decreased significantly after mixing cooked vegetable with FO peanut sauce (19.58%) or RO peanut sauce (6.23%) (Table 4). The reduction could be because of the decrease in the amount of combined cooked vegetables after addition of peanut sauce resulting in a reduction in antioxidant activity (1 : 1 ratio or 150 g : 150 g) and/or an increase in the number of free radicals from the addition of FO- or RO peanut sauce. This is consistent with the decrease in antioxidant activity due to the addition of peanut sauce (Table 3). During frying, peanuts can undergo lipid oxidation because of the high levels of unstable polyunsaturated fatty acids (PUFAs; linoleic and linolenic acids). PUFAs are susceptible to lipid peroxidation, which leads to the production of free radicals and peroxides [24]. Free electron build-up depletes existing antioxidants and, therefore, reduces their capacity to inhibit lipid peroxidation.

### 3.5. Estimation of Nutrition Content of Pecel Salad

We estimated the nutritional value of Pecel salad in our study (Material and Methods) based on the proximate composition available from Indonesian Food Composition Data (https://nilaigizi.com/) as presented in Table 5. The nutrition details in 100 g of ready-to-eat Pecel salad are 138.5 calories, 5.5 g protein, 11.1 g carbohydrate, and 7.0 g fat.

Following the guide of “My-plate” (“isi piringku”) from the Ministry of Health, Republic of Indonesia, regarding portions of a healthy diet, one-third of the plate must be vegetables (all kinds). The standard average requirement for teenagers and adults per day is 400–600 g mixed ready-to-eat vegetables. One portion of Pecel salad (cooked vegetable) is approximately equal to 300–500 g (depending on the portion size for each individual) of vegetables. As the moisture content of raw and cooked Pecel vegetables is similar (Table 1), one portion of Pecel salad dressed with peanut sauce can fulfil the recommended portion for a healthy diet with a higher antioxidant contribution compared to that from the raw vegetables.

## 4. Conclusions

The results from this study demonstrated that to achieve the highest antioxidant activity, the Pecel vegetables, green leafy vegetables, flowery vegetables, and mung-bean sprouts, should be boiled briefly for 1 min, water-steamed for 1 min, or water-blanched for 20 seconds. Seasoned peanut sauce for Pecel should be prepared by frying peanuts in fresh palm oil and seasoned with fresh garlic and lime leaves, as has been the decade-old practice. Seasoned peanut sauce using raw peanuts fried in repeatedly used frying oil significantly reduced the antioxidant activity and the inhibition of lipid peroxidation. The results of this study can be used to make recommendations on the cooking methods to preserve the health benefits of Pecel vegetables.

## Figures and Tables

**Table 1:** Effect of cooking methods on the moisture content of cooked Pecel vegetables.

Vegetables	Moisture content (%)				*p*
	Raw	Blanching^a^	Boiling^b^	Steaming^c^	
Turi flower	94.39 ± 0.68^a^	92.24 ± 0.57^b^	92.02 ± 0.01^b^	$\underset{¯}{93.36\pm 0.21}$ ^c^	0.019^∗^
Spinach leaf	90.56 ± 0.24^a^	85.63 ± 1.49^b^	$\underset{¯}{90.26\pm 0.16}$ ^a^	87.53 ± 0.11^c^	0.008^∗^
Papaya leaf	87.40 ± 0.42^a^	82.66 ± 1.06^b^	$\underset{¯}{87.83\pm 0.03}$ ^a^	84.31 ± 0.18^c^	0.002^∗^
Kenikir leaf	87.76 ± 0.43^a^	81.96 ± 1.05^b^	$\underset{¯}{87.42\pm 0.33}$ ^a^	88.77 ± 0.11^c^	0.001^∗^
Yard-long beans	90.88 ± 0.18	90.79 ± 2.52	$\underset{¯}{90.03\pm 0.03}$	91.43 ± 0.14	0.754
Mung-bean sprouts	82.08 ± 0.08	$\underset{¯}{83.82\pm 2.11}$	79.19 ± 2.11	79.99 ± 0.19	0.110
	Raw	Cooked (underlined)			*p*
Mixed vegetables	88.84 ± 3.98	88.83 ± 2.78			0.990

Values are presented as the average ± SD of 2 replicates. ^*p*^One-Way ANOVA with ^∗^*p* values were significant at *p* < 0.05. ^a^Blanching for 20 s, ^b^boiling for 1 min, and ^c^steaming for 1 min. The underlined values are the vegetables selected according to Table 2 (with the highest antioxidant activity) for subsequent analyses of combined cooked vegetables.

**Table 2:** Effect of cooking methods on antioxidant activity of Pecel vegetables.

Vegetables	Antioxidant activity (%)				*p* values
	Raw	Blanching^a^	Boiling^b^	Steaming^c^	
Turi flower	33.20 ± 5.95^a^	32.77 ± 3.26^a^	53.84 ± 2.59^b^	$\underset{¯}{60.25\pm 3.63}$ ^b^	<0.001^∗^
Spinach leaf	43.35 ± 3.82^a^	41.94 ± 6.11^a^	$\underset{¯}{43.15\pm 9.80}$ ^a^	20.88 ± 1.99^b^	0.005^∗^
Papaya leaf	33.67 ± 3.62^a^	12.60 ± 11.90^b^	$\underset{¯}{59.04\pm 5.35}$ ^c^	22.20 ± 2.62^b^	<0.001^∗^
Kenikir leaf	46.49 ± 1.07^a^	16.27 ± 1.49^b^	$\underset{¯}{54.93\pm 6.32}$ ^c^	20.66 ± 1.45^b^	<0.001^∗^
Yard-long beans	79.66 ± 5.01^a^	59.07 ± 3.58^b^	$\underset{¯}{70.21\pm 8.91}$ ^a^	55.05 ± 1.32^b^	0.002^∗^
Mung-bean sprouts	24.37 ± 2.35^a^	$\underset{¯}{49.27\pm 3.69}$ ^b^	10.18 ± 2.13^c^	22.85 ± 3.78^a^	<0.001^∗^

All data are presented as the mean ± SD (*n* = 3). *p* values by one-way ANOVA. ^∗^Significant when *p* < 0.05. ^a^Blanching for 20 seconds. ^b^Boiling for 1 minute. ^c^Steaming for 1 minute. The underlined numbers were the selected vegetables for subsequent analyses of combined cooked vegetables.

**Table 3:** Antioxidant activity of peanuts, peanut sauce, and combined cooked Pecel vegetables without or with peanut sauce.

Antioxidant activity	Percentage Mean ± SD	^∆^Reduction in antioxidant activity	*p* values	*P* values
(a). Peanut (raw and fried in fresh (FO) or repeatedly used oil (RO))				
Fresh raw peanut	44.45 ± 5.75	-	0.004^∗^	
FO fried peanuts	34.65 ± 1.06	9.8		
RO fried peanuts	27.53 ± 2.36	16.92		

(b). Fried peanut compared to peanut sauce				
FO fried peanuts	34.65 ± 1.06	-		0.023^∗^
FO peanut sauce	48.07 ± 6.39	-		
RO fried peanuts	27.53 ± 2.36	-		0.037^∗^
RO peanut sauce	34.77 ± 3.33	-		

(c). Peanut sauce				
FO peanut sauce	48.07 ± 6.39	-		0.033^∗^
RO peanut sauce	34.77 ± 3.33	-		

(d). Combined cooked vegetables without or with peanut sauce				
Combined vegetables^abc^	61.11 ± 2.27	-	<0,001^∗^	
Pecel FO	42.74 ± 4.22	18,37		
Pecel RO	38.80 ± 2.99	22.31		

All data are presented as mean ± SD (*n* = 3). ^*p*^One-way ANOVA, ^∗^significant when *p* < 0.05. ^*P*^Independent *t*-test, significant when *p* < 0.05. ^a^Papaya leaves, kenikir leaves, spinach, and yard-long beans were boiled for 1 min. ^b^Turi flowers water-steamed for 1 minute. ^c^Bean sprouts blanched in boiling water for 20 seconds. *∆* = reduction of antioxidant activity (FO peanut: fresh raw peanut; RO peanut: fresh raw peanut). Pecel FO: combined cooked vegetables with FO peanut sauce; Pecel RO: combined cooked vegetables with RO peanut sauce.

**Table 4:** Inhibition of lipid peroxidation by combined cooked Pecel vegetables with or without peanut sauce.

Treatment	Mean ± SD	*p* value
Combined vegetables^a,b,c^	23.81 ± 0.65	<0.001^∗^
Pecel FO	19.58 ± 3.6	
Pecel RO	6.23 ± 0.83	

All data are presented as the mean ± SD (*n* = 3). ^*p*^One-way ANOVA. ^∗^Significant when *p* < 0.05. ^a^Papaya leaves, kenikir leaves, spinach, and yard-long bean stewed for 1 min. ^b^Turi flowers steamed for 1 minute. ^c^Bean sprouts blanched in boiling water for 20 s. Pecel FO: combined cooked vegetables with FO peanut sauce; Pecel RO: combined cooked vegetables with RO peanut sauce.

**Table 5:** Nutrition content of Pecel vegetables based on the database of Indonesian Food Composition List (DKBM) (INA)^∗^.

Food	Weight (g)	Energy (cal)	Protein (g)	Carbohydrate (g)	Fat (g)
Pecel vegetables (raw)					
Turi flower	25	10.5	0.5	1.52	0.15
Spinach leaf	25	5.75	0.3	0.92	0.15
Papaya leaf	25	21.75	2	2.975	0.5
Kenikir leaf	25	11.25	0.92	1.65	0.12
Yard-long beans	25	7.5	0.57	1.45	0.1
Mung-bean sprouts	25	9.25	1.1	0.95	0.12
Total (a)	150	66	5.39	9.46	1.14
Peanut sauce					
Fried peanut	50	282	12.75	12.75	22.2
Palm sugar	30	110.4	0	13.8	0
Garlic	5	5.6	0.22	1.06	0.01
Water	100	-	-	-	-
Total (b)	185	398	12.97	27.61	22.21
Total a+b	335	464	18.36	37.07	23.35
Pecel salad	100	138.5	5.5	11.1	7.0

^∗^INA: TKPI/DKBM 2019/https://nilaigizi.com/.

## Data Availability

All data have been placed in manuscript.
